# Theoretical Study of Charge Mobility in Crystal Porphine and a Computer Design of a Porphine-Based Semiconductive Discotic Liquid Mesophase

**DOI:** 10.3390/ijms24010736

**Published:** 2023-01-01

**Authors:** Liana Savintseva, Alexander Avdoshin, Stanislav Ignatov, Alexander Novikov

**Affiliations:** 1 Department of Chemistry, Lobachevsky State University of Nizhny Novgorod, 23 Gagarin Ave, 603950 Nizhny Novgorod, Russia; 2 Institute of Chemistry, Saint Petersburg State University, Universitetskaya Nab., 7/9, 199034 Saint Petersburg, Russia; 3 Infochemistry Scientific Center, ITMO University, Kronverksky Pr., 49, Bldg. A, 197101 Saint Petersburg, Russia

**Keywords:** charge transport, charge mobility, transfer integral, organic semiconductors, neuromorphic material, liquid crystal, TD-DFT

## Abstract

Organic semiconductors are the focus of numerous studies; they are used in electronic devices. Modern research involves the production of neuromorphic organic materials, including those based on liquid crystal materials. The purpose of this work involves the theoretical modeling of molecules (the “core with branches” type) to construct a discotic mesophase capable of performing the functions of a neuromorphic material. For this purpose, the conductivity of crystal porphine, which can act as the nucleus of a molecule of the “core with branches” type, was investigated. The Marcus theory charge mobility values for the hole and electron were 0.148 and 0.088 cm^2^/V·s, respectively (the MOO method for calculating transfer integrals), and 0.561 and 0.160 cm^2^/V·s (DIPRO method). Based on TD-HF (HF-3c level of theory) calculations, possible structures of molecules for the formation of a discotic mesophase are proposed.

## 1. Introduction

In the past few decades, organic semiconductors (OS) have been the focus of numerous studies, supported by the confidence that one day they will replace (or at least compete with) more traditional inorganic materials [1,2]. This confidence is based on the relative simplicity of the specific adaptations of their properties by chemical syntheses, the potential low costs of their mass production, and the possibility of using wet processes in the manufacturing of devices. One of the modern directions of the OS study involves the creation of neuromorphic materials based on memristors, organic synaptic transistors, optical neuromorphic materials, and phase-change memory (PCM) devices [3,4,5,6]. The least studied direction involves the creation of material where intra- and intermolecular charge transfers will act as signals (pulses) of the nervous tissues of animals. Such material, if we consider it as a computing system (artificial neural network, ANN), would allow us to achieve a high density of computational elements, three-dimensional organization, and high computational efficiency.

Charge mobility is the most important parameter in many semiconductor applications [7]. As with most macroscopic properties, charge mobility strongly depends on the properties of macromolecular aggregation, i.e., it is a phase rather than a molecular property, although the specific molecular packaging will depend on the molecular structure [8]. Organic crystals tend to form crystalline “powders”, where homogeneous order is present within each domain. Each grain boundary makes a mess, and this is the place where the charge carrier slows down or even delays, which seriously affects the observed overall mobility of the charge. With some materials, it is difficult to obtain ordered crystals large enough to be tested in research laboratories, let alone obtained on a production line, where experimenters have to invent complex and often time-consuming protocols to obtain them.

It may be reasonable to compromise (losing order while maintaining manufacturability). This can be achieved by using liquid crystals (LCs), where the orders of three-dimensional solids are only partially lost, and for the same reason, larger uniformly ordered samples are usually achievable. In LCs formed by disk-shaped molecules, the charge transfer is the most consistent and promising result [2]. To function as an ANN, the molecules of the material must have high values of charge mobility; the ability to adapt to intermolecular contacts depends on the type of signal, which can be achieved in a disk-shaped LC.

**Figure 1:**
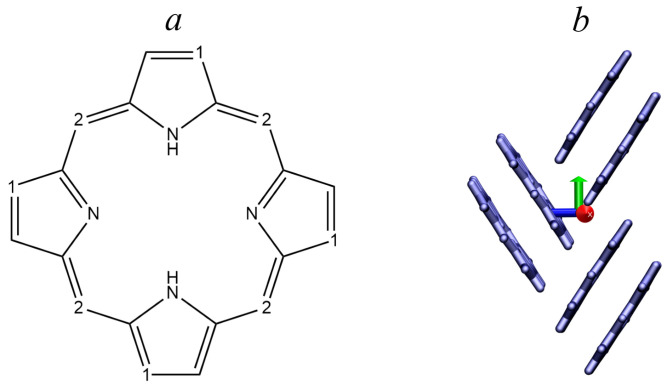
(**a**) Porphine molecule with two possible positions of the substituents (marked as 1 and 2). (**b**) Fragment of porphine crystal packaging with axis directions.

As model molecules, it is reasonable to consider porphine and its derivatives, which (i) participate in the electron transfer process in biological systems; (ii) are promising semiconductors that are actively studied; (iii) can act as the “core” of a discotic liquid crystal. Porphine (Figure 1) is a tetrapyrrole compound, an organic semiconductor involved in the charge transfers in many biological systems. In recent years, it has been studied as a fragment of organic field-effect transistors [9,10,11], solar cells [12,13,14], liquid crystals for modern electronics applications [15,16], and a fragment of organometallic framework compounds [17,18,19]. It was shown in [9] that porphine-based molecules can form “head-to-tail” structures, demonstrating a relatively high mobility of carriers in a direction parallel to the aromatic ring, which is important for our study.

In addition to focusing on novel organic semiconductors with good mobility performances, understanding the relationship between molecular structures, crystal packing, and device performance remains a great challenge for chemists and material scientists.

Thus, in this work, we studied the conductivity of crystalline porphine and determined the charge mobility for the electron and the hole. It was shown that despite the small values of the reorganization energy for the electron, the hole conductivity prevails in the crystal, which is typical for organic semiconductors. The influences of some substituents on the electronic properties of porphine were analyzed. Based on this, a model of a molecule of the “core with branches” type was proposed, which can form a liquid crystal phase under the condition of elongation of hydrocarbon chains and, theoretically, will meet the listed requirements for neuromorphic material.

## 2. Theory and Methods

Charge mobility describes how fast an electron and a hole can move from molecule to molecule in the bulk of a material. The process of the hole and electron transfer between identical molecules can be expressed as D${}^{+}$···A → D···A${}^{+}$/D${}^{-}$···A → D···A${}^{-}$, respectively. Charge mobility is defined as the ratio of the absolute value of the drift velocity to the absolute value of the intensity of this field, i.e., the mobility of the charge is always non-negative. Thus, the main task is to determine the drift velocity of the charge carrier.

The traditional approach to determining the charge transfer rate is the high-temperature limit of the classical theory, i.e., the Marcus theory [20], where the transfer rate constant is given by the analytical expression

(1) $$\begin{array}{cc} \omega_{ij} & =\frac{2\pi }{\hbar }\frac{J_{ij}^{2}}{\sqrt{4\pi \lambda_{ij}k_{B}T}}exp\left[-\frac{{\left(\Delta E-\lambda_{ij}\right)}^{2}}{4\lambda_{ij}k_{B}T}\right], \end{array}$$

where $k_{B}$ is the Boltzmann constant, $J_{ij}$ is the electronic coupling element or transfer integral, $\lambda_{ij}$ is the reorganization energy. It was originally formulated to address outer sphere electron transfer reactions in solutions, in which the two chemical species only change in their charge with electron jumping, but do not undergo large structural changes. It was extended to include inner sphere electron transfer contributions, in which a change of distances or geometry in the coordination shells of the two chemical species is taken into account. Currently this method is widely used for prediction charge mobility in organic semiconductor materials [1,21,22].

The transfer integral is a measure of the strength of the electronic coupling of the frontier orbitals of monomers mediated by the dimer interactions. It is usually evaluated using an effective one-particle Hamiltonian:

(2) $$\begin{array}{c} J_{ij}=\langle \varphi_{i}\left|\hat{H}\right|\varphi_{j}\rangle . \end{array}$$

$\varphi_{i}$ and $\varphi_{j}$ are diabatic wave functions localized, respectively, on molecules *i* and *j* involved in the charge transfer reaction, and $\hat{H}$ is the Hamiltonian of the formed dimer (charge transfer complex). Transfer integrals are very sensitive to the distance and mutual orientation of the molecules involved in the charge transfer. Thus, they are significantly affected by static and/or dynamic disordering, so it is very important to explicitly calculate $J_{ij}$ for each “jumping” pair in its realistic morphology.

There are two approaches used to estimate transfer integrals: MOO (molecular orbital overlap), which does not require a self-consistent calculation for a dimer and is based on Zerner’s intermediate neglect of differential overlap (ZINDO) Hamiltonian, and DIPRO (the projection of monomer orbitals on a manifold of explicitly calculated dimer orbitals), which includes both calculations for monomers and dimers [23]. An approximate method based on ZINDO is substantially faster than the first principle approaches, since it avoids the self-consistent calculations of each individual monomer and dimer. This allows constructing the matrix elements of the INDO Hamiltonian of the dimer from the weighted overlap of molecular orbitals of the two monomers. However, MOO is based on the ZINDO Hamiltonian, which has limited applicability [24]. The general advice is to first compare the accuracy of the MOO method to the DFT-based calculations.

These methods are in good agreement with each other [24]; however, there is evidence that the first one reproduces better the values of the transfer integrals during the rotation of molecules relative to each other [23], so we performed calculations using both methods.

The reorganization energy takes into account the change in nuclear degrees of freedom due to the movement of the charge from the donor to the acceptor. Its physical meaning is that it represents the energy spent on “tuning” the molecular environment of the donor and acceptor to make a “jump” between them. There are two contributions to the reorganization energy: intramolecular, which is caused by the rearrangement of the nuclear coordinates of two molecules forming a charge transfer complex, and extraspheric, which is caused by the relaxation of the nuclear coordinates of the medium and the change in the polarization of the surrounding molecules.

For the cases involving the discharge (i.e., loss of an electron) of molecule *i* and the charge of molecule *j*: $\lambda_{ij}^{int}=\lambda_{i}^{cn}+\lambda_{j}^{nc}=U_{i}^{nC}-U_{i}^{nN}+U_{j}^{cN}-U_{j}^{cC}$; here, $U_{i}^{nC}$ is the internal energy of neutral molecule *i* in the geometric structure of its charged state (a small *n* denotes the ground state and a large *C* denotes the structure of the charged molecule). Similarly, $U_{j}^{cN}$ is the energy of a charged molecule *j* in the geometry of the neutral state. The potential energy surfaces of the donor and acceptor are not identical for chemically different compounds or conformers of the same molecule; therefore, in the general case, $\lambda_{i}^{cn}\neq \lambda_{j}^{nc}$.

In the present work, the Gaussian 09 (Revision C.01) package was used for the monomer and dimer orbitals calculations; all other DFT calculations were carried out using the ORCA [25,26,27] suite with the M062X functional [28] in conjugation with the cc-pVTZ basis set [29]. This combination (M062X/cc-pVTZ) has proven itself well for determining the electronic properties of organic semiconductors [30]. And TD-HF calculations were carried out utilizing the HF-3c method [31], which reproduces well the qualitative spectral picture of porphine and its derivatives (see SI). Charge transfer simulations were carried out in the VOTCA-CTP package [32,33,34]. Transfer integrals by DIPRO were analyzed via the CATNIP [35] program and the NTO analysis was performed in TheoDORE [36,37].

## 3. Results

### 3.1. Conductivity of Crystalline Porphine

The crystal structure of porphine (a = 10.2262 Å, b = 11.9060 Å, c = 12.3853 Å, Z = 4, and α = 90°, β = 101.711°, γ = 90°, ρ = 1.396 g/cm${}^{3}$) and its metal derivatives have been well studied experimentally [38]. Molecular geometries of neutral and ionized states, as well as electronic properties, such as ionization potential (IP), electron affinity (EA), and reorganization energies, were calculated. The reorganization energy for electron transfer is half the energy of the hole reorganization in Table 1.

**Figure 2:**
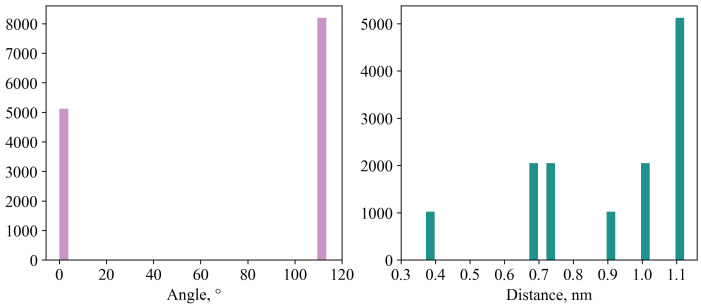
Distribution of angles between normals to the porphine planes in a crystal and distances between the geometric centers of molecules.

All possible pairs of molecules in the crystal fragment (2048 molecules) were determined using the minimum distance between the atoms of the molecules (0.4 nm) and divided into groups based on the values of the angles between the ’normals’ and the planes of the porphine ring (*a*) and the distances between the geometric centers of the molecules (*d*) (Figure 2). As a result, 13,312 pairs of molecules were detected in our system, and 7 possible variants of the mutual orientations of the molecules in the crystal package were obtained: d=0.37, a=0.0; d=0.68, a=113.0; d=0.73, a=113.0; d=1.12, a=113.0; d=0.92, a=0.0; d=1.01, a=0.0; d=1.12, a=0.0 (*d* in nm and *a* in degrees. For a more accurate estimation of the transfer integrals, the geometry obtained by the DFT method was substituted into a dimer taken from a crystal (so-called mapping).

**Figure 3:**
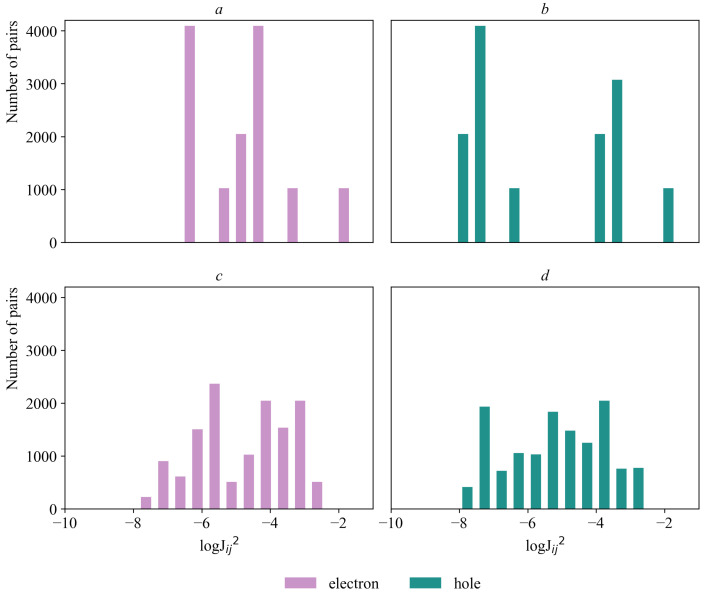
Distribution of transfer integrals in crystalline porphine: (**a**,**b**) DIPRO method; (**c**,**d**) MOO method.

The transfer integrals were evaluated by two methods: MOO in the VOTCA-CTP software package and DIPRO in the CATNIP program; moreover, the Hamiltonian of the semi-empirical ZINDO method was used (Figure 3).

After the list of neighboring molecules (neighbor list) was determined and all necessary parameters (atomic charges, reorganization energies, and transfer integrals) were calculated, the charge transfer in a crystal fragment (2048 molecules) was simulated using the kinetic Monte Carlo algorithm (KMC). During the simulation, the field was superimposed along each axis and the charge mobility was averaged over 10 trajectories (Table 2).

Table 2 shows that the mobilities predicted by both methods are consistent with each other in some directions. In the X–Y–Z direction, they rise and fall together, except for the electron mobility in the X direction. The electron mobility is maximal in the X direction for the MOO method (red axis, Figure 1b) and the Y direction for the DIPRO method, and the hole mobility is in the Y direction (green axis, Figure 1b) in both methods. The mobility of the hole is slightly greater than the mobility of the electron in the MOO method and noticeably higher in the DIPRO method. Thus, the hole conductivity prevails over the electronic one, despite the fact that the reorganization energy for an electron is half of that for a hole. Experimental charge mobilities for metallo-octaethylporphyrins (M-OEP) are from 0.014 cm^2^/V·s for Pd-OEP to 0.200 cm^2^/V·s for Co-OEP [11], which allows at least to estimate the reliability of the results obtained. For crystalline porphine without substituents, we found no data on experimentally or theoretically determined charge mobilities.

### 3.2. The Effects of Substituents in the Porphine Ring

Porphine has a crystalline structure and is usually a powder where the long-range order is only present within one domain. To obtain a discotic liquid mesophase, it is necessary to add mobile extended substituents to the flat porphine ring. In order to select such substituents, it is important to know exactly how different types of substituents affect the electronic properties of the molecule. For this purpose, the following substituents were analyzed: phenyl as an aromatic radical, allyl as an unsaturated hydrocarbon radical, and normal butyl as a limiting hydrocarbon radical. The influences of different fragments on the electronic properties were evaluated, as well as the influences of the locations in the porphine rings.

All derivatives were semiconductors with band gaps from 4.5 eV for the porphine molecule to 4.0 eV for the tetra-allyl-porphine molecule at the substituent 2 (TAP-2) position (Table 3). Adiabatic ionization potentials (IP) and electron affinity (EA) were determined. The IP decreased slightly for all derivatives and reached a minimum value of 6.3 eV for the tetra-butyl-porphine molecule at the position of substituent 2 (TPP-2); EA increased slightly for TPP and TAP molecules and decreased for TnBP. Lower IP values favor electron transfer since the transfer process is preceded by electron energization from HOMO to LUMO.

**Figure 4:**
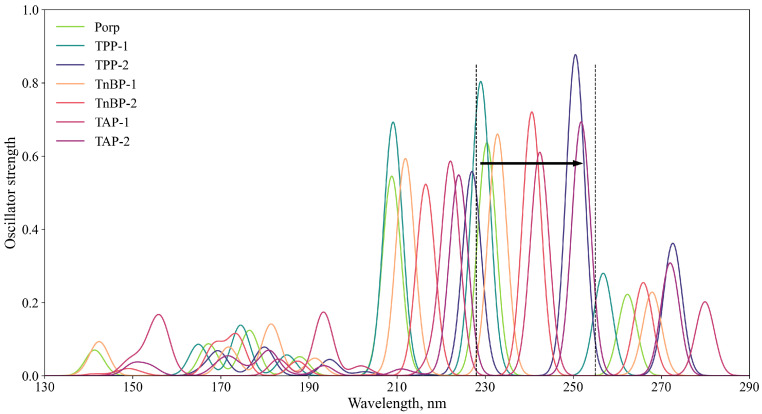
Excitation spectra of porphine derivatives obtained by TD-HF (HF-3c level of theory, full width at half maximum (FWHM) = 5 nm). The arrow shows the shift of the main excitation on the red side from TPP-1 to TAP-2 and the dashed lines separate the three main excitations, which are typical for porphine.

The excitation spectra for the first 30 adiabatic excitations of all porphine derivatives under consideration were obtained by the TD-HF method (Figure 4). The calculation was performed by the HF-3c method, which reproduced the qualitative spectral pictures of more expensive methods well, but worked out much faster (see SI). In sequence TPP-1–porphine–TnBP-1–TnBP-2–TAP-1–TPP-2–TAP-2, the spectrum shifted to the red side, while no qualitative changes were observed; all molecules had typical spectral patterns for porphine (Figure S1). Thus, we found that various substituents in the porphine ring lowered the ionization potential and the excitation energy, but did not change the nature of the excitation itself, which is useful when creating a conductive liquid crystal material.

It was shown that the device fabricated from the spin-coating film of metal-free tetra(phenyl)porphine (TPP-2 in our notation) exhibited a carrier mobility for a hole as high as 0.007 cm${}^{2}$/Vs [43]. Field-dependent mobility was also evidenced with values reaching 0.012 cm${}^{2}$/Vs. The devices fabricated from the tetrakis(4-pentyloxyphenyl)porphine (TPOPP-2 in our notation) single crystal displayed a relatively good OFET performance with the carrier mobility for a hole 1.8 × 10${}^{-3}$ cm^2^/Vs in the direction parallel to the aromatic porphyrin ring [9]. Since we have not found any experimental studies for the other substitutes, it is difficult to rely on these data to correlate with our results. However, these studies show that porphine derivatives can demonstrate good values of charge mobility (hole mobility), including along the plane of the porphine ring, which is consistent with our conclusions.

### 3.3. Dependence of the Value of the Transfer Integral on the Mutual Orientation of Molecules

**Figure 5:**
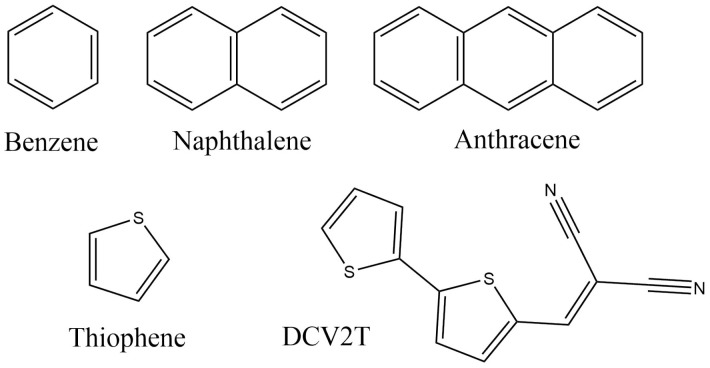
Candidate molecules for the creation of neuromorphic material based on porphine.

Discotic liquid crystals are essentially structures of the “core with branches” type, where the maximum values of charge mobility, as a rule, are achieved with the vertical movements of charge carriers along stacks of flat nuclei. However, when creating neuromorphic material, the movement of the charge “from the tail to the nucleus” is more interesting because it resembles the movement of an impulse through the nervous tissue. The next task is to select a “tail” (i) that would have a large overlap integral with the porphine ring; (ii) for which the rate of charge transfer to porphine would be significantly greater than the inverse; (iii) for which virtual NTOs would be centered. As candidate molecules for the role of the “tail”, flat π-conjugated molecules—known as organic semiconductors—were selected (Figure 5).

**Figure 6:**
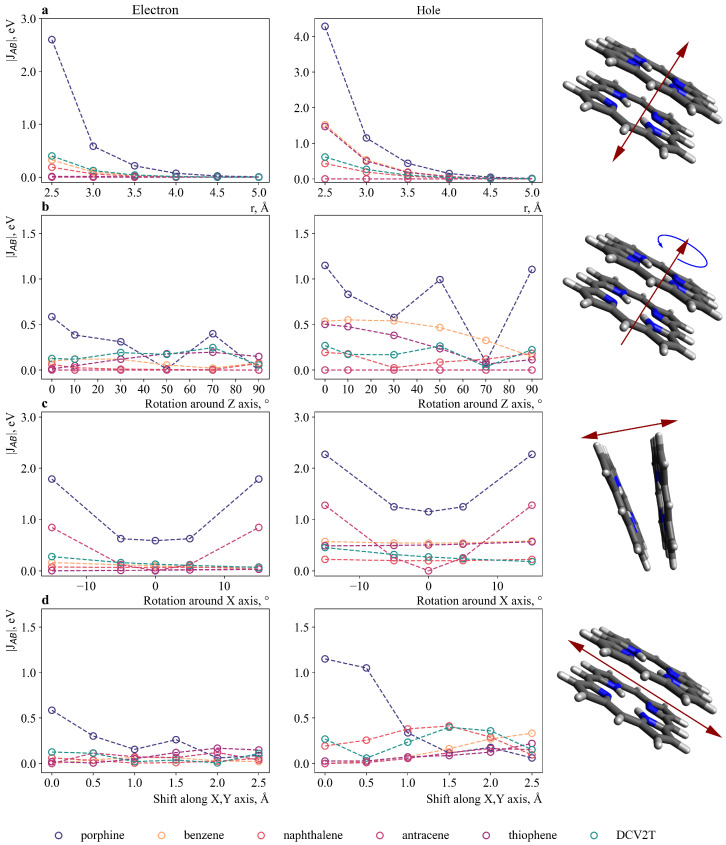
Change in transfer integrals between porphine and another molecule depending on: (**a**) the distance between geometric centers; (**b**) the magnitude of the angle of rotation around the normal (r = 3 Å); (**c**) the magnitude of the angle of the molecule plane’s inclination relative to the plane of porphine (r = 3 Å); (**d**) the magnitude of the shift in molecules relative to each other (r = 3 Å). Note: small molecules (benzene and thiophene) were placed above the center of the five-membered ring, and not above the center of porphine (for (**a**–**c**)).

For porphine molecules and selected molecules, it is important to estimate the values of the transport integrals over the entire spaces of possible mutual orientations to have an idea about what kind of conductivity will be in an amorphous material (for example, in an LC), and not in a crystal, where the number of mutual orientations is very limited. The molecules were placed in such a way that their planes were parallel to each other, and the geometric centers were strictly one above the other. The following parameters were changed in turn: the distance between the geometric centers (Figure 6a); the angle of rotation around the normal to the plane of the ring (Figure 6b); the angle of inclination of the plane of one molecule to another (Figure 6c); the shift in the plane of the molecules relative to each other (Figure 6d).

The values of the transfer integrals for holes are (on average) twice as large as those of electrons for all molecules, which suggests that in all such materials the hole’s conductivity will prevail over the electronic one. The transfer integrals for two porphine molecules were significantly larger than those for porphine (for any of the molecules under consideration). For thiophene and benzene molecules, the integrals were large only if the molecules were located clearly above the five-membered porphine ring, and decreased sharply with displacement (Figure 6d). The transfer integrals for an anthracene molecule depend sharply on orientation; for example, when the angle of rotation relative to the axis lying in the plane of the molecule (here, we call it the x-axis) changes from 0 to 15 degrees, the value of the transfer integral increases from 0 to 1270 meV (Table S3). This behavior makes it difficult to analyze this molecule. Therefore, DCV2T, which has sufficiently large transfer integrals over the entire studied space of mutual orientations, seems to be the most interesting at this stage.

## 4. Discussion

**Figure 7:**
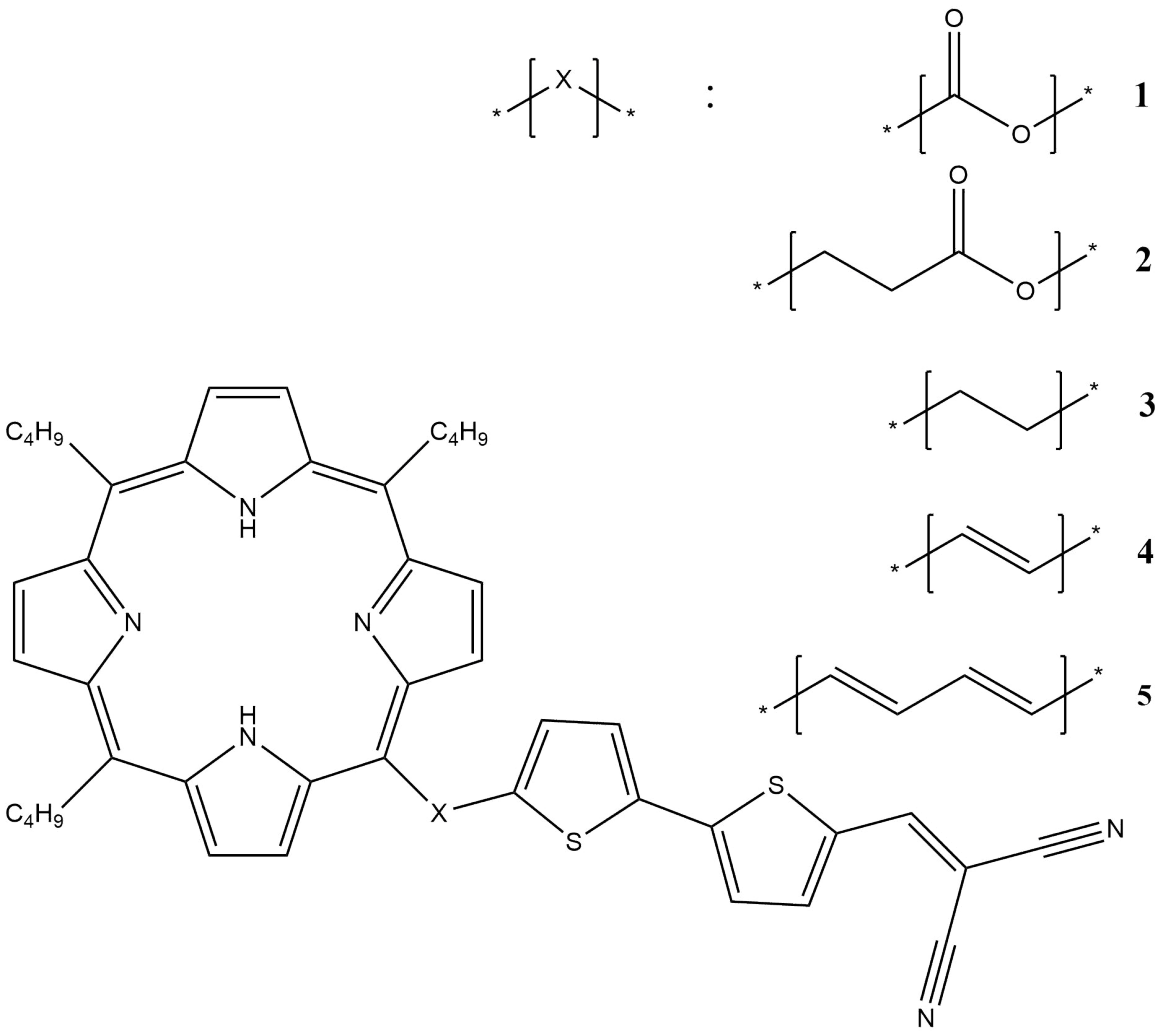
Model structures.

Based on the results, it is possible to propose model structures of molecules of the “core with branches” type, from which a liquid crystal mesophase can be obtained. The lengths of the peripheral substituents, of course, should be longer, but for simplicity of calculations, we focus on butyl. Since DCV2T turned out to be the most interesting (with the highest values of transfer integrals when changing the mutual orientation, with the exception of non-parallel orientations), TnBP-based structures are proposed, where one butyl is replaced by DCV2T. At the same time, different variants of the addition of molecules were investigated (Figure 7).

**Figure 8:**
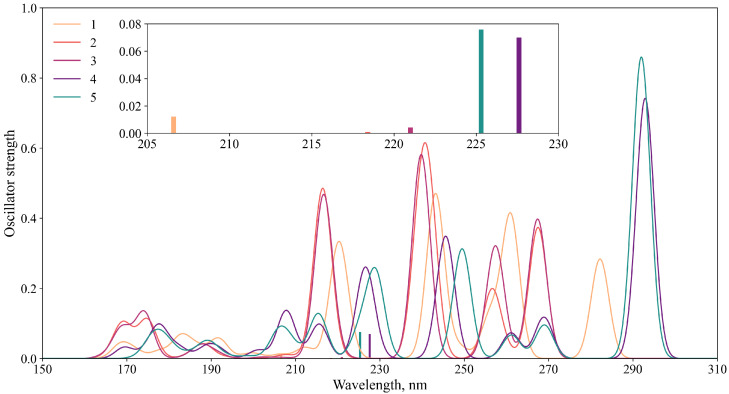
Excitation spectra of model structures, FWHM = 5 nm. Sticks represent excitations with CT contributions.

**Figure 9:**
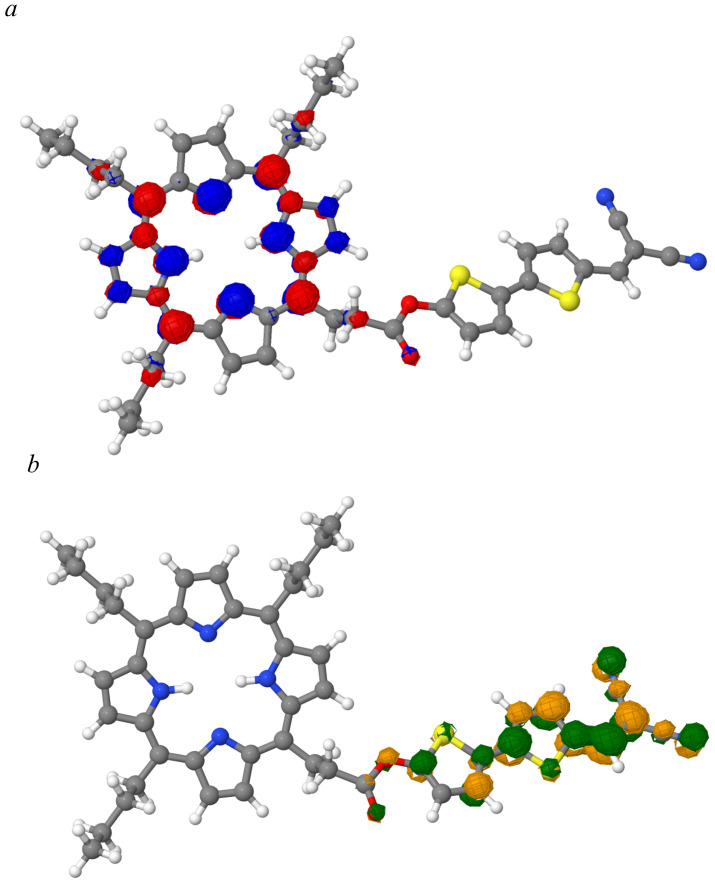
Ninth excitation of molecule **2** with a full separation of NTO into two fragments. (**a**) Occuped NTO. (**b**) Virtual NTO.

Attachment via -CH${}_{2}$-CH${}_{2}$-C(O)O- is borrowed from nature: for example, a phytol molecule is attached to chlorophyll, and the rest are taken in order to diversify the options under study. As already mentioned, it would be interesting to obtain a molecule in which the occupied NTO is localized on the porphine nucleus, and the virtual NTO is located on the tail. To do this, the TD-HF calculation was made by the same method HF-3c (Figure 8). In terms of local molecular orbitals and rigid fragments, all of the main excitations in such systems were local (an electron and a hole were distributed on one fragment—nucleus or tail). We were interested in states with charge transfer (CT), i.e., when the electron density moves from one fragment to another. There are such states but they are poorly expressed. For molecules **1**–**3**, such excitations are single configurations (fully separated), but for molecules **4**–**5**, they are not, and they have extra contributions (double configurations) (Table S4). An example of a fully separated excitation of molecule 2 is given in Figure 9 (ninth excitation from Table S4).

Thus, the excitations we are interested in have very low intensities. It was interesting to see which electronic properties from these molecules manifested in the liquid crystal phase, because the influence of the environment and the violation of the long-range order and symmetry will undoubtedly lead to changes in electronic properties.

Although the estimates obtained within the Marcus model and TD-HF theory allows obtaining only approximate picture, the macroscopic parameters (such as charge mobility, energy disorder, and photoexcitation) charge separation anyway, giving the guiding tool for the prediction of the required properties of semiconductive organic mesophases. The achieved representation can be further improved by adding the effects of the environment or the long-range disorder or symmetry violation, which undoubtedly leads to changes in electronic properties.

## 5. Conclusions

To tailor new organic semiconductor materials, we considered several molecular models based on substituted porphyrins designed for charge transfer in a liquid crystal mesophase medium with branched elements. To estimate the charge mobility in such a mesophase, the Marcus theory was used with the evaluation of the transfer integrals by the MOO and DIPRO methods, which gave similar results. The methods used were verified using a model based on a porphyrin dimer with an experimental crystalline phase structure. For this model, charge mobility estimates were 0.148 cm^2^/V·s (MOO) and 0.088 cm^2^/V·s (DIPRO) for electrons, and 0.160 cm^2^/V·s (MOO) and 0.561 cm^2^/V·s (DIPRO) for holes, in satisfactory agreement with experimental data. The dimer model made it possible to study in detail the effects of structural distortions of various types on the values of the transfer integrals. Five compounds were studied as substituted porphyrin derivatives, including those with substituents selected on the basis of natural compounds. It was shown that, regardless of the structure of the porphine-based material (crystal, film, or amorphous material), the hole conductivity will prevail over the electronic one due to the fact that the transfer integrals for the holes are almost twice as large as for the electrons.

Three designed models with optical photoexcitation produced states with separated charges. The most promising compounds seem to be model molecules **2** and **3**, in which at the excitation energies of 5.677 and 5.609 eV, electrons and holes separated (14.9 and 13.1 Å, respectively). Since these values were obtained by the HF-3c method, the obtained estimates of excitation energies should be considered as upper boundaries for experimental values.

## Figures and Tables

**Table 1:** Hole and electron reorganization energies for the porphine molecule obtained by the M062X/cc-pVTZ level of theory.

Carrier Type	$\lambda_{1}$, eV	$\lambda_{2}$, eV
Hole	0.15	0.15
Electron	0.07	0.07

**Table 2:** Charge mobility in cm^2^/V·s with standard deviation, obtained by the KMC method (averaged over 10 launches), superimposed field 10${}^{6}$ V.

Method	Carrier Type	X ^1^	Y ^1^	Z ^1^
MOO	electron	0.148 ± 0.006	0.013 ± 0.003	0.006 ± 0.002
	hole	0.036 ± 0.005	0.160 ± 0.006	0.127 ± 0.006
DIPRO	electron	0.004 ± 0.001	0.088 ± 0.005	0.007 ± 0.002
	hole	0.097 ± 0.007	0.561 ± 0.011	0.172 ± 0.007

^1^ The axis where the field is superimposed.

**Table 3:** Effects of substituents and their positions in the porphine ring on adiabatic IP, adiabatic EA, and the band gap.

Substituent	Position No from Figure 2	Designation	IP, eV	EA, eV	Band Gap, eV
-H	1, 2	Porp	7.0 (6.6–6.9) ^1^ [39]	1.5	4.5
-phenyl	1	TPP-1	6.6	1.6	4.4
	2	TPP-2	6.9 (6.3–6.4) ^1^ [40,41]	1.7 (1.7) ^1^ [42]	4.2
-allyl	1	TAP-1	6.7	1.7	4.2
	2	TAP-2	6.5	1.8	4.0
n-butyl	1	TnBP-1	6.8	1.4	4.4
	2	TnBP-2	6.3	1.5	4.1

^1^ Experimental values.

## Data Availability

Not applicable.

## Supplementary Materials

The following supporting information can be downloaded at https://www.mdpi.com/article/10.3390/ijms24010736/s1.

## Abbreviations

The following abbreviations are used in this manuscript:

MOO	molecular orbital overlap
DIPRO	dimer projection
KMC	kinetic Monte Carlo
IP	ionization potential
EA	electron affinity
TPP	tetraphenylporphine
TAP	tetra-allyl-porphine
TnBuP	tetra-n-butyl-porphine
DCV2T	dicyanovinyldithiophene
CT	charge transfer
