# Supplementary material for: Theoretical Study of Charge Mobility in Crystal Porphine and a Computer Design of a Porphine-Based Semiconductive Discotic Liquid Mesophase
Source: Int J Mol Sci. 2023 Jan 1;24(1):736. doi: 10.3390/ijms24010736 (PMC9821175; doi:10.3390/ijms24010736)
Supplement: Supplementary file 1 [file ijms-24-00736-s001.zip › ijms-2087140-supplementary.pdf]

Supplementary Information

The M062X/cc-pVTZ level of theory reproduces well geometric and electronic properties of organic semiconductors, which was demonstrated by us in the previous work [1]. However, it is sensitive to the integration grid, which makes this method quite expensive. Therefore, the M062X method was compared with the HF-3c composite approach in ORCA program for porphine molecule. It was shown that HF-3c reproduces well the qualitative picture of the excitation spectrum (Figure S1), while the spectrum shifts to the violet region, and the intensity increases. Other excitation characteristics are also reproduced correctly, such as the degree of multi-configuration of the excitation, the distance between the electrons and the hole (Table S1), as well as the view of NTO (Figure S2). Also, both methods reproduce well the typical picture of the excitation spectra of porphyrins.

Table S1. Analysis of excited states of porphine based on NTO was performed in the TheoDore program [2-3]. The first 10 excitations are given. dE is the excitation energy in eV, f is the oscillator strength, PR_{NTO} is the degree of multi-configuration of excitation (how many NTO are involved in each excitation), RMShe is the root-mean square electron-hole separation or exciton size in Å

State	dE (eV)	f	PR_{NTO}	RMShe
		M062X/cc-pVTZ VT*		
1(1)A	2.420	0.004	2.055	4.573
2(1)A	2.634	0.004	2.046	4.657
3(1)A	4.007	0.730	1.872	4.412
4(1)A	4.066	0.000	1.162	4.307
5(1)A	4.318	1.799	2.144	4.662
6(1)A	4.512	0.000	1.126	4.839
7(1)A	4.641	0.557	1.216	4.874
8(1)A	4.666	1.922	1.766	4.800
9(1)A	4.737	0.000	1.133	3.834
10(1)A	4.769	0.000	1.140	3.832
		HF-3c VT*		
1(1)A	2.727	0.007	2.036	4.202
2(1)A	3.073	0.005	2.128	4.286
3(1)A	4.918	1.085	2.256	4.196
4(1)A	5.015	0.000	1.307	3.877
5(1)A	5.556	3.487	2.129	4.542
6(1)A	5.868	0.000	1.302	3.327
7(1)A	5.958	0.019	1.315	3.296
8(1)A	6.069	3.080	1.779	4.485
9(1)A	6.141	0.000	1.226	3.521

10(1)A	6.243	0.000	1.251	3.482
HF-3c AT*				
1(1)A	2.541	0.001	2.080	4.176
2(1)A	2.914	0.014	2.127	4.264
3(1)A	4.727	1.184	2.265	4.194
4(1)A	4.838	0.000	1.308	3.851
5(1)A	5.381	3.387	2.118	4.511
6(1)A	5.648	0.000	1.347	3.228
7(1)A	5.718	0.019	1.358	3.201
8(1)A	5.939	2.901	1.703	4.438
9(1)A	6.012	0.000	1.579	4.242
10(1)A	6.069	0.000	1.228	3.513

*VT – vertical transition, AT – adiabatic transition

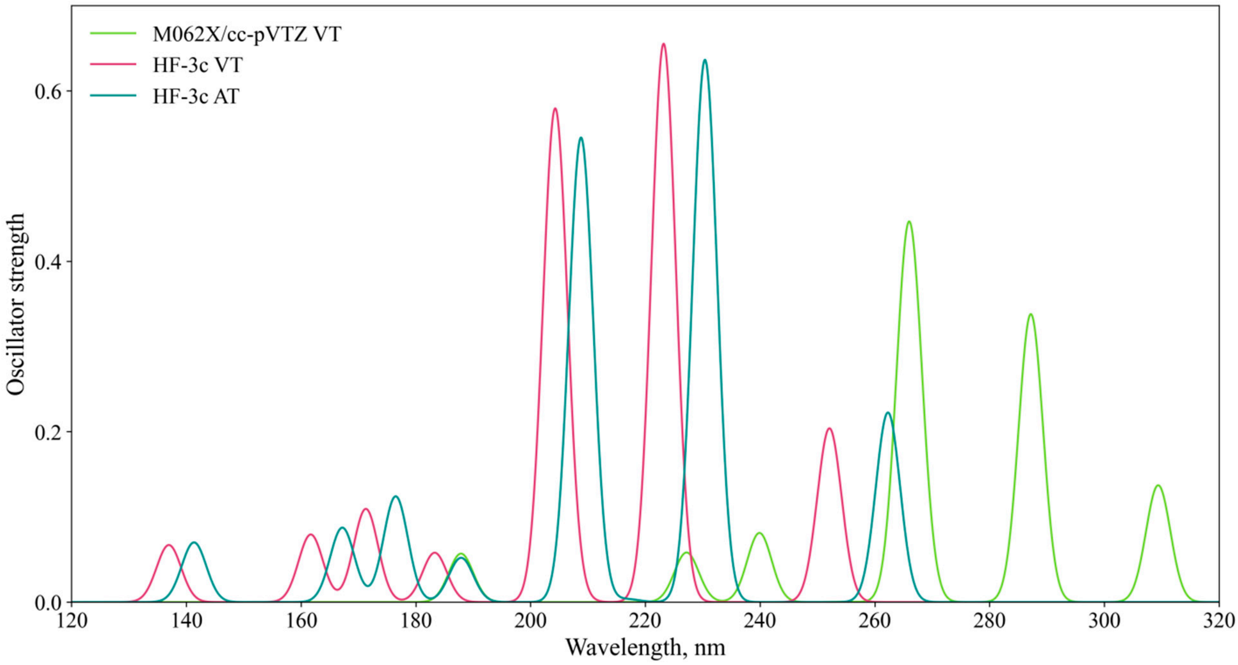

Figure S1. Comparison of porphine excitation spectra obtained by TD-DFT (30 excited states) using different methods, full width at half maximum (FWHM) = 5 nm

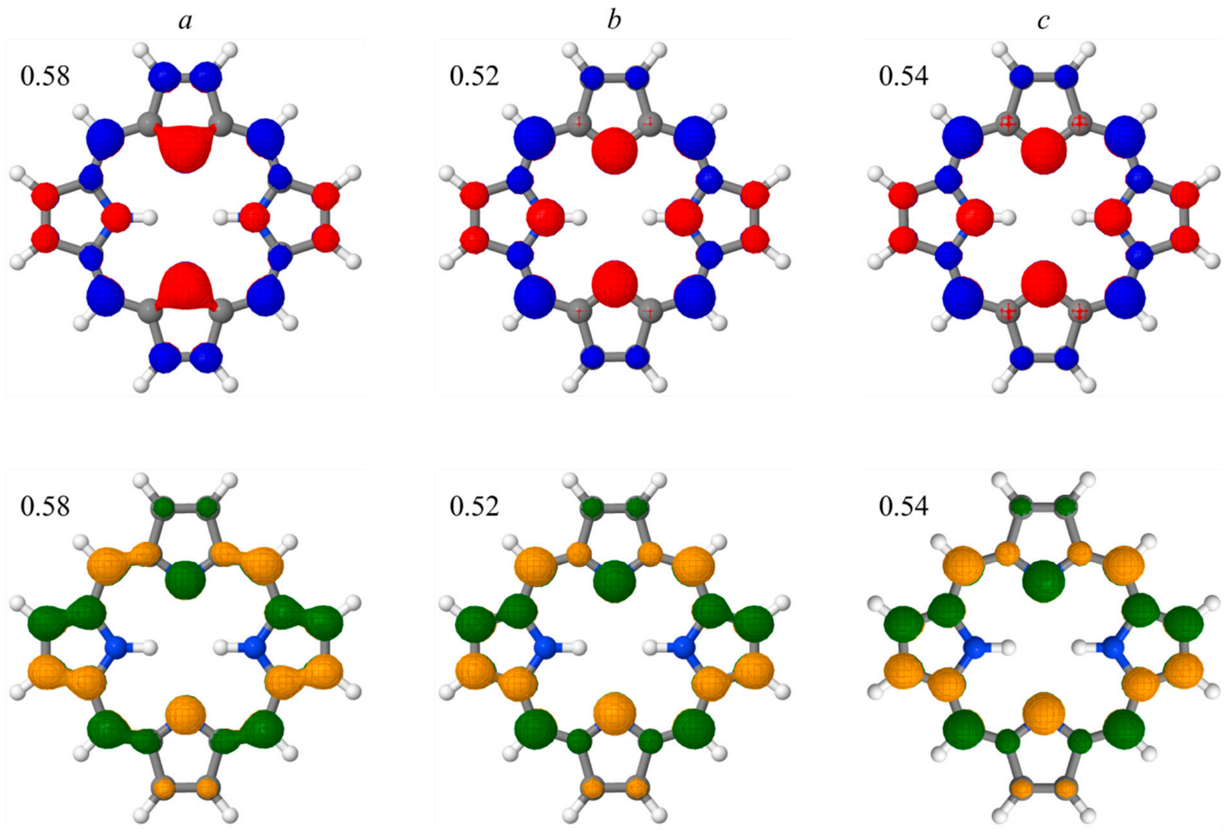

Figure S2. View of NTO porphine with the greatest contribution to the fifth excitation TD-DFT: *a* - M062X/cc-pVTZ VT, *b* - HF-3c VT, *c* - HF-3c AT. Red/blue - occupied orbitals, yellow/green - virtual orbitals. The contribution of this NTO to the fifth excitation is indicated on each orbital

Similar results were obtained for the tetraphenylporphine molecule (Table S2, Figure S3, Figure S4)

Table S2. Analysis of excited states of tetraphenylporphine based on NTO. The first 10 excitations are given

State	dE (eV)	f	PR_{NTO}	RMShe
M062X/cc-pVTZ VT				
1(1)A	2.298	0.007	2.012	4.893
2(1)A	2.497	0.017	2.029	4.991
3(1)A	3.790	1.613	2.249	4.827
4(1)A	3.949	0.000	1.192	4.464
5(1)A	3.953	2.969	2.294	5.174
6(1)A	4.297	1.608	1.471	5.143
7(1)A	4.388	0.000	1.159	5.070
8(1)A	4.456	0.122	1.075	5.207
9(1)A	4.604	0.000	2.102	5.433

10(1)A	4.649	0.000	1.881	6.005
HF-3c VT				
1(1)A	2.621	0.050	1.965	4.390
2(1)A	2.959	0.019	2.131	4.455
3(1)A	4.769	1.824	2.331	4.404
4(1)A	4.930	0.000	1.343	3.940
5(1)A	5.182	4.696	2.231	4.790
6(1)A	5.698	3.201	1.714	4.590
7(1)A	5.861	0.000	1.307	3.422
8(1)A	5.950	0.015	1.323	3.404
9(1)A	6.100	0.000	1.679	4.515
10(1)A	6.114	0.000	1.331	3.632
HF-3c AT				
1(1)A	2.460	0.046	1.984	4.438
2(1)A	2.796	0.017	2.148	4.504
3(1)A	4.549	1.925	2.332	4.474
4(1)A	4.711	0.000	1.351	3.951
5(1)A	4.949	4.669	2.242	4.884
6(1)A	5.461	2.968	1.660	4.650
7(1)A	5.658	0.000	1.356	3.356
8(1)A	5.728	0.015	1.369	3.328
9(1)A	5.811	0.000	1.980	4.568
10(1)A	5.937	0.000	2.072	4.637

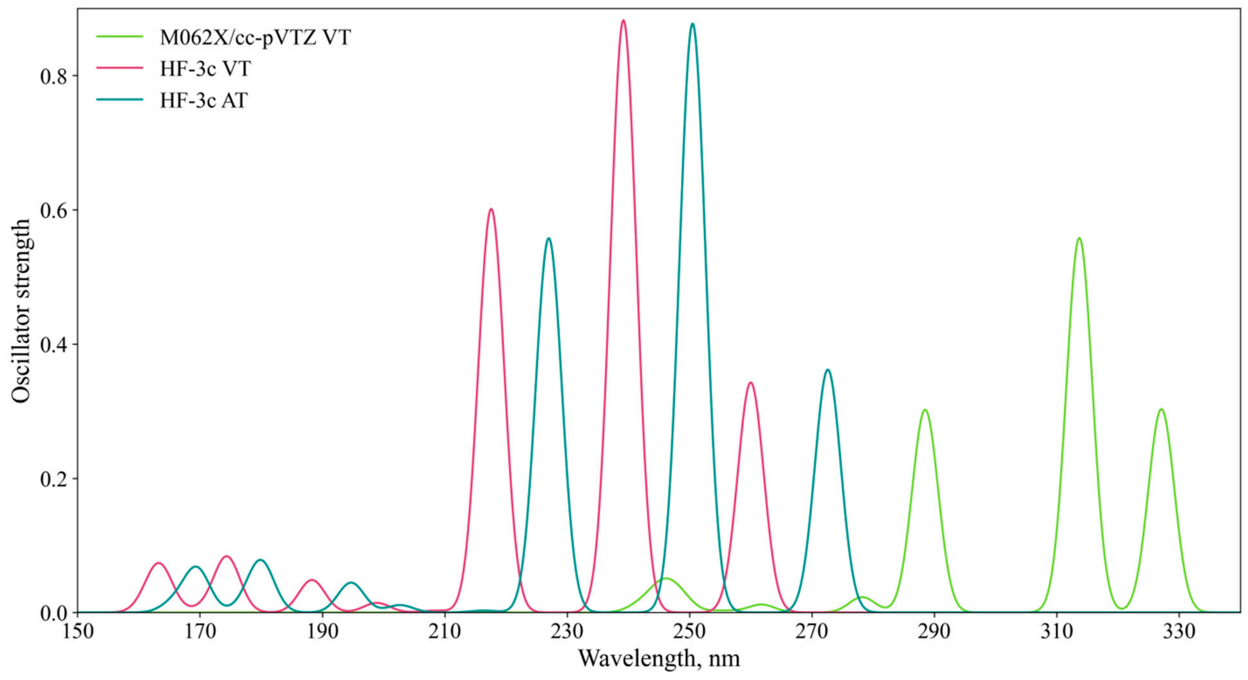

Figure S3. Comparison of tetraphenylporphine excitation spectra obtained by TD-DFT (30 excited states) with different methods, FWHM = 5 nm

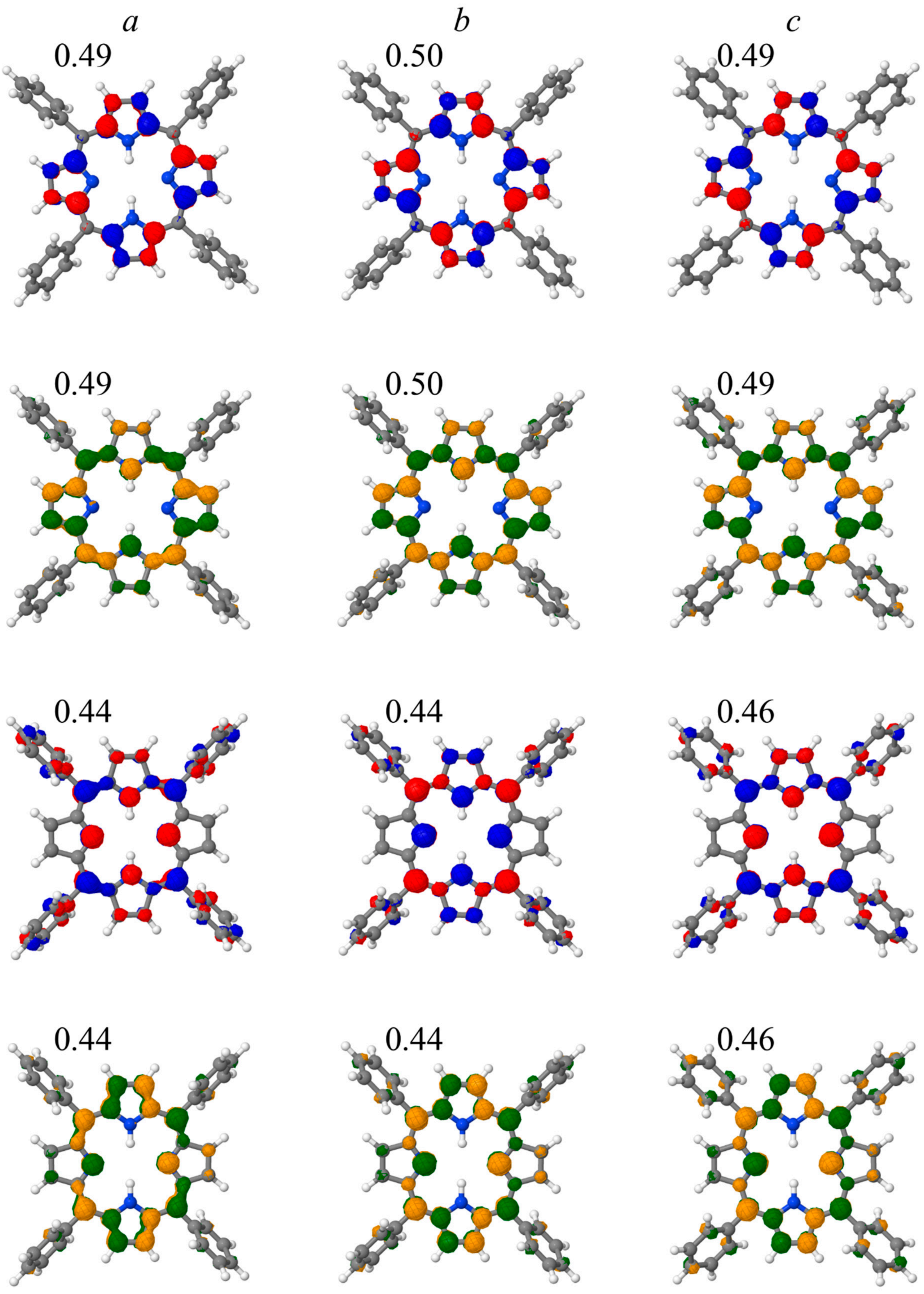

Figure S4. View of two porphine NTO with the greatest contribution to the fifth TD-DFT excitation: *a* - M062X/cc-pVTZ VT, *b* - HF-3c VT, *c* - HF-3c AT. Red/blue - occupied orbitals, yellow/green - virtual orbitals. The contribution of this NTO to the fifth excitation is indicated on each orbital

The Table S3 shows the values of the modulus of the transfer integrals (in meV) for the studied molecules in different variants of mutual orientation.

Table S3. The values of the modulus of transfer integrals in meV for porphine with different molecules when the distances between the geometric centers of the molecules (r), the angle of rotation relative to the normal to the porphine plane (z), rotation relative to the axis lying in the porphine plane (x) and the shift of the molecules relative to each other (xy) were changed.

Charge carrier	Molecule	**r, Å**					
		2.5	3.0	3.5	4.0	4.5	5.0
e	Porphine	2602.7	585.5	215.8	73.6	23.3	6.8
	Benzene*	326.0	95.5	37.5	13.5	4.5	1.4
	Napthalene	187.7	62.4	18.8	5.2	1.3	0.3
	Antracene	1.3	0.2	0.1	0.0	0.0	0.0
	Thiophene*	13.2	12.8	6.0	2.1	0.7	0.2
	DCV2T	401.3	126.5	40.8	12.7	3.7	1.0
h	Porphine	4283.0	1149.4	437.9	151.9	48.3	14.2
	Benzene*	1522.3	536.2	198.3	67.7	21.3	6.2
	Napthalene	432.4	193.0	73.7	25.5	8.1	2.4
	Antracene	1.2	0.2	0.0	0.0	0.0	0.0
	Thiophene*	1470.8	501.9	182.0	61.6	19.2	5.5
	DCV2T	614.7	268.3	105.4	37.6	12.2	3.6
		**z, °**					
		0	10	30	50	70	90
e	Porphine	585.5	385.0	310.4	10.8	398.3	0.0
	Benzene*	95.5	121.5	118.0	55.6	20.5	80.5
	Napthalene	62.4	25.3	9.2	6.0	4.7	72.2
	Antracene	0.2	0.2	0.1	0.2	0.0	0.0
	Thiophene*	12.8	46.8	119.6	178.8	195.0	148.0
	DCV2T	126.4	118.7	191.7	171.6	246.6	558.7
h	Porphine	1149.4	831.4	577.3	993.3	85.2	1104.9
	Benzene*	536.2	550.8	540.1	466.5	325.4	146.4
	Napthalene	193.0	178.6	24.9	86.4	119.9	181.4
	Antracene	0.2	0.0	0.3	0.0	0.2	0.2
	Thiophene*	501.9	476.3	380.5	234.9	65.8	113.2
	DCV2T	268.3	171.7	167.7	263.6	35.9	223.5
		**xy, Å**					
		0	0.5	1.0	1.5	2.0	2.5
e	Porphine	585.5	302.4	155.3	262.1	61.1	88.8
	Benzene	0.0	41.1	66.7	63.4	29.6	21.9
	Napthalene	62.4	34.7	4.8	10.8	26.4	57.0
	Antracene	0.2	115.8	74.2	65.8	120.9	38.9

	Thiophene	20.2	6.00	52.5	120.9	168.7	148.9
	DCV2T	126.4	113.1	21.0	38.7	7.3	111.7
h	Porphine	1149.4	1050.0	336.9	115.2	177.7	61.8
	Benzene	0.0	25.1	73.2	163.1	271.1	332.5
	Napthalene	193.0	256.6	380.1	413.1	289.9	95.5
	Antracene	0.2	10.8	56.1	118.0	165.0	152.3
	Thiophene	27.1	27.8	72.5	88.1	125.8	220.8
	DCV2T	268.3	59.6	234.9	398.6	359.3	152.6

		**x, °**				
		-15	-5	0	5	15
e	Porphine	1788.5	624.6	585.5	624.6	1788.3
	Benzene*	158.0	115.6	95.5	75.7	33.0
	Napthalene	71.5	63.0	62.4	63.0	71.5
	Antracene	843.2	117.9	0.2	118.3	843.7
	Thiophene*	2.7	7.7	12.8	18.0	30.6
	DCV2T	274.7	157.1	126.4	104.3	63.3
h	Porphine	2271.2	1247.2	1149.4	1247.3	2271.3
	Benzene*	567.6	540.1	536.2	540.8	579.7
	Napthalene	220.8	196.5	193.0	196.4	220.8
	Antracene	1276.8	251.8	0.2	252.4	1278.2
	Thiophene*	486.4	491.8	501.9	517.4	563.9
	DCV2T	451.3	315.3	268.3	231.9	177.8

* small molecules (benzene and thiophene) were placed above the center of the five-membered ring, and not above the center of porphine

Table S4. Analysis of excited states based on NTO of model molecules: states with charge transfer contribution are given.

Molecule	State	dE (eV)	f	PR_{NTO}	RMShe
Molecule1	12(1)A	6.000	0.065	1.124	11.559
	16(1)A	6.373	0.001	1.468	11.010
Molecule2	9(1)A	5.677	0.005	1.008	14.902
	16(1)A	6.177	0.000	1.009	14.917
Molecule3	7(1)A	5.609	0.022	1.027	13.132
	13(1)A	6.123	0.001	1.893	11.061
Molecule4	7(1)A	5.447	0.372	1.539	9.575
Molecule5	8(1)A	5.503	0.403	1.995	11.366

References

1. Savintseva, L.A., Avdoshin, A.A. & Ignatov, S.K. Charge Transport in Biomimetic Models of Organic Neuromorphous Materials. *Russ. J. Phys. Chem. B* 16, 445–454 (**2022**). https://doi.org/10.1134/S1990793122030216
2. J. Chem. Phys. **152**, 084108 (**2020**); https://doi.org/10.1063/1.5143076
3. J. Chem. Phys. 141, 024106 (**2014**); https://doi.org/10.1063/1.4885819
